# Autoencoder-Inspired Convolutional Network-Based Super-Resolution Method in MRI

**DOI:** 10.1109/JTEHM.2021.3076152

**Published:** 2021-04-28

**Authors:** Seonyeong Park, H. Michael Gach, Siyong Kim, Suk Jin Lee, Yuichi Motai

**Affiliations:** 1 Department of BioengineeringUniversity of Illinois at Urbana-Champaign 14589 Urbana IL 61820 USA; 2 Department of Radiation OncologyWashington University in St. Louis 7284 St. Louis MO 63130 USA; 3 Department of Radiation OncologyDivision of Medical PhysicsVirginia Commonwealth University 6889 Richmond VA 23284 USA; 4 TSYS School of Computer ScienceColumbus State University 2634 Columbus GA 31907 USA; 5 Department of Electrical and Computer EngineeringVirginia Commonwealth University 6889 Richmond VA 23284 USA

**Keywords:** Autoencoder, convolution neural network, deep learning, MRI, super resolution

## Abstract

Objective: To introduce an MRI in-plane resolution enhancement method that estimates High-Resolution (HR) MRIs from Low-Resolution (LR) MRIs. Method & Materials: Previous CNN-based MRI super-resolution methods cause loss of input image information due to the pooling layer. An Autoencoder-inspired Convolutional Network-based Super-resolution (ACNS) method was developed with the deconvolution layer that extrapolates the missing spatial information by the convolutional neural network-based nonlinear mapping between LR and HR features of MRI. Simulation experiments were conducted with virtual phantom images and thoracic MRIs from four volunteers. The Peak Signal-to-Noise Ratio (PSNR), Structure SIMilarity index (SSIM), Information Fidelity Criterion (IFC), and computational time were compared among: ACNS; Super-Resolution Convolutional Neural Network (SRCNN); Fast Super-Resolution Convolutional Neural Network (FSRCNN); Deeply-Recursive Convolutional Network (DRCN). Results: ACNS achieved comparable PSNR, SSIM, and IFC results to SRCNN, FSRCNN, and DRCN. However, the average computation speed of ACNS was 6, 4, and 35 times faster than SRCNN, FSRCNN, and DRCN, respectively under the computer setup used with the actual average computation time of 0.15 s per $100\times100$

## I. Introduction

Magnetic Resonance Imaging (MRI) has superior soft tissue contrast versus x-ray-based imaging techniques such as Computed Tomography (CT) and cone beam CT [1]. MRIs can be acquired continuously without the risks of ionizing radiation. Therefore, MRI-guided Radiation Therapy (MRIgRT) is preferred for treating tumors that are affected by motion, including lung tumors located near critical or radiosensitive organs i.e. organs at risk (OARs) such as the esophagus, heart, or major vessels [1]. Currently, a cine of a single image plane containing the tumor is acquired to gate radiation dose delivery in MRIgRT. However, treatment gating is desired using the entire tumor volume and neighboring OARs to minimize errors associated with through-plane tumor motion. Thus, real-time four-dimensional (4D) MRI is being developed for MRIgRT. 4D MRI typically suffers from low spatial resolution (e.g., ≥ 3.5 *mm* in-plane resolution) due to the constraints of k-space acquisition, temporal resolution, and system latency [2]. Therefore, new techniques are required to optimize the spatial and temporal resolution of 4D MRI in MRIgRT.

Real-time 4D acquisitions are being accelerated using under-sampled non-Cartesian k-space trajectories and compressed sensing or iterative image reconstruction methods [3], [4]. Unfortunately, these techniques require computationally intensive and time-consuming image reconstruction algorithms [3]. Super-Resolution (SR) is a potential solution for the Low spatial Resolution (LR) problem [5]–[12]. Specifically, there is a demand for recovery of missing resolution information on each slice of MRI, which is considered an in-plane resolution problem [8].

Deep learning-based single image SR methods have been recently introduced in computer vision [9]–[12]. Deep learning is a new breakthrough technology that is a branch of machine learning [13], [14]. Many existing deep learning studies have addressed various applications such as classification, detection, tracking, pattern recognition, image segmentation, and parsing. They have also demonstrated robust performance of deep learning compared to other machine learning tools. In medical imaging, many deep learning-based frameworks have been introduced for feature extraction, anatomical landmark detection, and segmentation [15]–[17]. Recently, deep learning-based single image SR methods for medical imaging have been actively explored [18]–[28].

In this paper, we propose an in-plane SR method for MRI, called Autoencoder-inspired Convolutional Network-based SR (ACNS) and investigate the relationship between the network architecture, i.e., intra-layer structure and depth, and its performance. The proposed method estimates High-Resolution (HR) MRI slices from the LR MRI slices according to a scaling factor. ACNS is composed of three steps: feature extraction, multi-layered nonlinear mapping, and reconstruction, where multiple nonlinear mapping layers have less nodes than the feature extraction and reconstruction layers. The intra-layer structure and depth of ACNS are empirically determined as a compromise between its qualitative performance and computational time.

The contributions of this paper are twofold. First, this study not only achieves high image quality of MRI but also significantly reduces SR computational time. Spatial resolution enhancement of MRI needs to be performed with minimal latency during MRIgRT. Thus, the most important problem in the use of the deep learning-based SR methods is overcoming their long computational time. This paper demonstrates potential application of the proposed method for in-plane SR of real-time 4D MRI in MRIgRT. Second, this study provides a detailed analysis of the relationship between the deep learning network architecture, i.e., intra-layer structure and depth, and its performance based on our experimental outcomes. The results suggest a developmental direction to forthcoming deep learning-based SR methods for the medical imaging.

## II. Related Works

### A. MRI Super-Resolution Methods

SR studies for MRI have been proceeding since 1997 [29]. There are two primary goals that the SR research has pursued: the in-plane resolution improvement [30]–[35] and the through-plane resolution improvement [36]–[42]. The in-plane resolution improvement is to remedy missing resolution information on 2D MRI, or a slice of 3D or 4D MRI. The through-plane resolution improvement is to reduce the slice thickness and remove aliasing between multiple slices of 3D or 4D MRI. The through-plane resolution for 3D acquisitions may be lower than the in-plane resolution for multi-slice 2D acquisitions [8]. Most studies have focused on through-plane resolution improvements. Recently, the in-plane resolution improvement methods for 4D MRI have been actively studied [20]–[28].

As shown in Table 1, the existing in-plane resolution improvement methods include an Iterative Back-Projection (IBP) [30], simple bilinear INTerpolation (INT), LASR, TIKhonov (TIK), Direct Acquisition (DAC), THEOretical curves (THEO) [32], Conjugated Gradients (CG) [33], Low-Rank Total-Variation (LRTV) [34], wavelet-based Projection-Onto-Convex-Set (POCS) SR [31], and Toeplitz-based iterative SR for improving Periodically Rotated Overlapping ParallEL Lines with Enhanced Reconstruction (PROPELLER) MRI [35], [43]. IBP is simple and fast, but is vulnerable to noise. Additionally, IBP has no unique solution for the SR problem due to the ill-posed nature of the inverse problem. Deterministic regularization-based methods, such as INT, LASR, TIK, DAC, THEO, CG, and LRTV, convert a LR observation model into the well-posed problem by using *a priori* information. However, the deterministic regularization-based methods are also vulnerable to noise. POCS methods (the wavelet-based SR and Toeplitz-based iterative SR) are robust for noisy and dynamic images, but their convergence speeds are slow and computational costs are high.

**TABLE 1:** In-Plane Super-Resolution of MRI

Category	Method	Limitation
Back-projection	Iterative Back-Projection (IBP)	Vulnerable to noise
Deterministic regularization	Simple bilinear INTerpolation (INT), LASR, TIKhonov (TIK), Direct Acquisition (DAC), THEOretical curves (THEO), conjugated gradients, and Low-Rank Total-Variation (LRTV)	Vulnerable to noise
Projection Onto Convex Sets (POCS)	Wavelet-based POCS SR and Toeplitz-based iterative SR	Slow convergence speed High computational cost

### B. CNN-Based Super-Resolution Methods

Various SR approaches have been introduced in computer vision. They can be categorized as reconstruction-based and learning-based SR methods. The reconstruction-based super resolution methods contain steering kernel regression [44] and nonlocal mean [45] algorithms. These approaches depend on prior knowledge such as total variation, edge, gradient profile, generic distribution, and geometric duality priors [46]–[50]. The learning-based SR methods include nearest neighbor [51], sparse coding [52], [53], Support Vector Regression (SVR) [54], random forest [55], joint [56], nonlinear reaction diffusion [57], conditional regression [58], Fourier burst accumulation-based method [59], and Convolutional Neural Network (CNN)-based methods [7]–[12], [60]. The learning-based methods map the relationship between the LR image and HR image so they can accurately recover missing details on the image. CNN-based SR methods in particular are state of the art [9], as they have shown superior image quality improvements, albeit at high computational cost.

The rapid advance of deep learning methods made a variety of the CNN-based SR methods applicable in medical images such as retinal images [8], [62] and MRIs [21]–[28]. Pham *et al.* [21], [22] applied the Super-Resolution Convolutional Neural Network (SRCNN) [9], [10] for brain MRIs. Chen *et al.* proposed a densely connected super-resolution network for brain MRIs in [23] that was inspired by a densely connected network [63]. Zhang *et al.* and Qui *et al.* proposed fast medical image SR for retina images [62] and efficient medical image SR for knee MRIs [24]. Both methods use an identical network structure with three hidden layers of SRCNN [9], [10] and a sub-pixel convolution layer proposed by Shi *et al.* [64]. Chaudhari *et al.* [25] [26] proposed DeepResolve for knee MRIs that consists of 20 layers of paired 3D convolution operator and a rectified linear unit. Zhao *et al.* [27] proposed a synthetic multi-orientation resolution enhancement method for brain MRIs using an Enhanced Deep Residual Network (EDRN) [65]. In [61], a SR Generative Adversarial Network (SRGAN) [66] was employed for the retinal images. For 4D MRI, Chun *et al.* proposed a cascaded deep learning method that consists of a denoising autoencoder [67], downsampling network, and SRGAN [66]. Most of the proposed methods of natural images have been adopted for the medical images with or without minor modifications in the network architecture or a combination of several methods [21], [23], [24], [28] [61], [62].

We compare our method to well-known CNN-based SR methods with a single network structure that are expected to provide fast computation speed for 4D MRI. Table 2 shows three major methods in CNN-based SR methods: SRCNN [9], [10]; Deeply-Recursive Convolutional Network (DRCN) [12]; and Fast SRCNN (FSRCNN) [11]. SRCNN is a basic form of CNN excluding a pooling process that was reformulated from the conventional sparse coding-based SR methods. SRCNN was used for brain MRI in [21], [22], [24], [62]. The test time of SRCNN was 0.39 s per image in dataset *Set14* [53] at the magnification power of 3. FSRCNN was redesigned from SRCNN with the additional process of shrinking and expanding to reduce its computational cost. The test time of FSRCNN was 0.061 s per image in dataset *Set14* at the magnification power of 3. DRCN has a partially recursive structure with an exceptionally connected component, called a ‘skip connection’ that is considered a form of ResNet [23], [61], [68]. The skip connection directly feeds input data into a reconstruction network. The computation time of DRCN was not measured in [12].

**TABLE 2:** Convolutional Neural Network-Based Super-Resolution

Method	Featured structure	Average Computation time
Super-Resolution Convolutional Neural Network (SRCNN)	No pooling process	0.39 s per image in dataset $Set14$ at magnification power of 3 [9]
Fast Super-Resolution Convolutional Neural Network (FSRCNN)	No pooling process and additional process of shrinking and expanding	0.061 s per image in dataset $Set14$ at magnification power of 3 [11]
Deeply-Recursive Convolutional Network (DRCN)	No pooling process, partially recursive layers, and skip connection	Not measured in [12]

## III. Methods & Materials

The aim of this study is to produce an HR MRI slice, i.e., larger matrix size with extrapolated signals, from an original MRI of $64\times64$ pixels or $128\times128$ pixels, according to a specified magnification power called the ‘scaling factor.’ For instance, the MRI generated by the scaling factor of ‘3’ would have a spatial resolution 3 times higher than the original MRI. When enlarging, i.e., upsampling, the original MRI, the image quality of the original MRI slice will naturally decrease without compensation for the absent resolution information. Although the original MRI has inadequate spatial resolution for use in anatomical MRI, edges shown in the MRI are sufficiently sharp. Blurring should not be neglected in LR images. Among downsampling methods, a bicubic interpolation results in blurry images rather than pixelated ones by calculating a weighted average of the nearest pixels. The result of the bicubic downsampling is highly analogous to the result from blurring with a $3\times 3$ average filter in addition to downsampling with a nearest-neighbor interpolation with the scaling factor of ‘2’. Thus, we model both pixel information loss and blurring caused by the bicubic downsampling as ACNS:

\begin{equation*}\boldsymbol {Y}=\boldsymbol {D}_{bicubic }^{f} \boldsymbol {X},\tag{1}\end{equation*}

where **Y** denotes an LR MRI corresponding to the original MRI, $\mathbf {D}_{bicubic}^{f}$ indicates the bicubic downsampling operator with a scaling factor $f$, and **X** is an HR MRI corresponding to the enlarged MRI that we desire to obtain. The proposed algorithm produces HR MRI from the original MRI **Y** with a scaling factor $f$ and a parameter set $\Theta $. We define an outcome of ACNS as the symbol **Z**. The lose function for the bicubic downsampling is produced from Eq. (1), as follows:

\begin{equation*} L_{D}\left ({\mathbf {Z} }\right)=\mathbf {Y}-\mathbf {D}_{bicubic}^{f}\mathbf {Z}\tag{2}\end{equation*}

The observation model is used to generate a training input dataset and the LR MRIs in our experiments.

With Eq. (2), we can translate the given image SR problem of MRI into an optimization problem:

\begin{equation*} \hat {\mathbf {X}}{=}\left \{{\underset {\mathbf {Z}}{\mathbf {argmin}}\left \|{ L_{D}\left ({\mathbf {Z} }\right) }\right \|^{\mathbf {2}} {:}\mathrm { \mathbf {Z}} {=}F {(}\mathbf {Y};f; {\Theta }) }\right \}\tag{3}\end{equation*}

where $\hat {\mathbf {X}}$ indicates an estimated HR MRI, **Z** is an outcome of ACNS, $F$($\cdot $) denotes the proposed method as a function, and $\Theta $ is a parameter set for $F$($\cdot $). The parameter set $\Theta $ includes filters (weights) and biases of each layer in ACNS.

**FIGURE 1.**
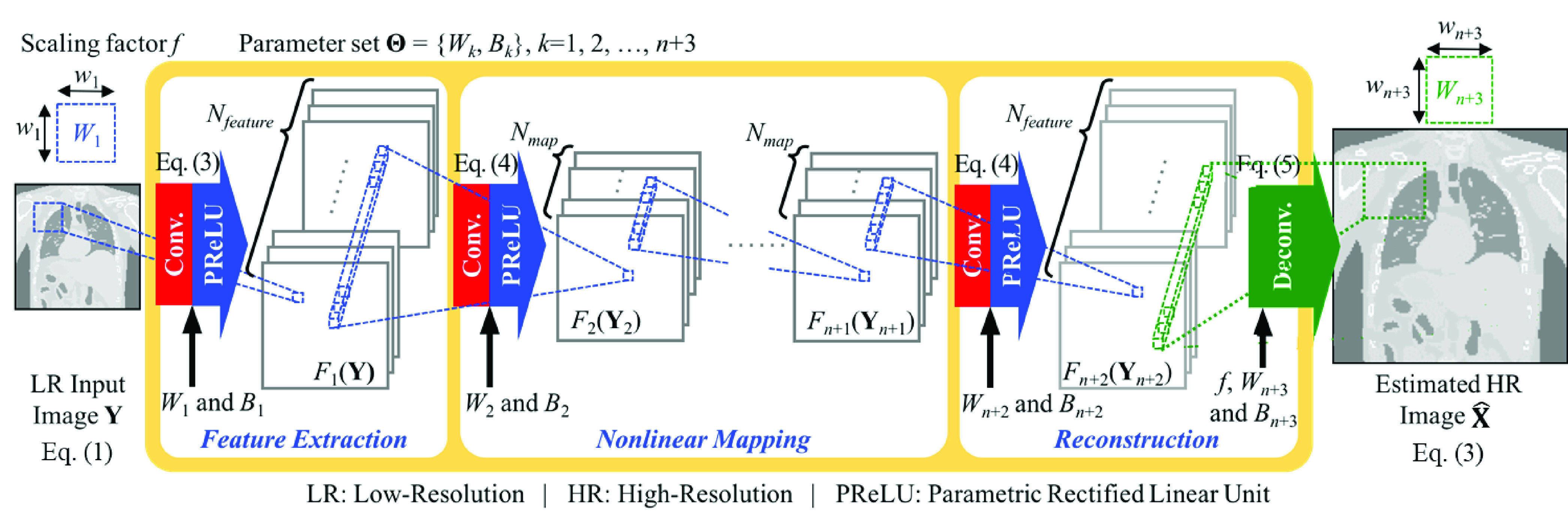
Autoencoder-inspired convolutional network-based super-resolution.

ACNS consists of convolution layers, activation function layers, and a deconvolution layer as illustrated in Fig. 1. Convolution layers and activation function layers are primary components of typical CNN. The other prime component of CNN is a pooling layer—also called a subsampling layer. The pooling layer chooses featured values from the image for progressive reduction of the number of parameters and the computational cost in the network, but it causes loss of input image information. In order to keep the feature values, the proposed method excluded the pooling layer in its architecture and designed ACNS with the deconvolution layer to upsample the LR MRI. We also determined the network structure of ACNS based on the experimental results.

ACNS was inspired by the autoencoder [69], one of the well-known artificial neural networks. The autoencoder achieves dimensionality reduction with less nodes in its hidden layers than those in the input and output layers. We hypothesized that critical features to be preserved through the network would be limited even though their exact number is unknown. Under this hypothesis, the structure of the autoencoder would perform well for the given SR problem if parameters are appropriately set for the network. Moreover, this structure would largely contribute to a decrease in test time by reducing the computation in the hidden layers of the network.

As shown in Fig. 1, ACNS is described as the three steps: feature extraction, nonlinear mapping, and reconstruction. In feature extraction, local features of **Y** are extracted depending on receptive fields as follows:

\begin{align*}F_{1} \left ({{{\mathbf { Y}}} }\right)=\max \left ({{W_{1} \ast {\mathbf { Y}}+B_{1},0} }\right)+\alpha \min \left ({{0,W_{1} \ast {\mathbf { Y}}\!+\!B_{1}} }\right),\!\!\! \\ {}\tag{4}\end{align*}

where an operator ‘*’ denotes a convolution, max(0, $\cdot$) + $\alpha $ min($\cdot $, 0) is an activation function, i.e., Parametric Rectified Linear Unit (PReLU) function [70], and $W_{1}$ and $B_{1}$ represent filters and biases of feature extraction operation, respectively. The size of $W_{1}$ is $w_{1}$, which restricts a unit range to extract the local features on **Y**. The number of $W_{1}$, i.e., $N_{feature}$, equals the number of the extracted features.

As existing studies proved, nonlinearity of mapping is a highly important process in CNN-based SR to enhance image quality performance [12]. Therefore, we iterate the nonlinear mapping between the **Y** and **X**:

\begin{align*}&\hspace {-1.5pc} F_{i+1} \left ({{{\mathbf { Y}}_{i+1}} }\right) \\=&\max \left ({{W_{i+1} \ast {\mathbf { Y}}_{i+1} +B_{i+1},0} }\right) \\&{ +\alpha \min \left ({{0,W_{i+1} \ast {\mathbf { Y}}_{i+1} +B_{i+1}} }\right), \quad 0< i\le n }\tag{5}\end{align*}

where $W_{i+1}$ indicates filters and $B_{i+1}$ represents biases of the $i$th recurrence of the nonlinear mapping operation. The number of $W_{i+1}$ is $N_{map}$. In Eq. (5), **Y**_2_ equals $F_{1}$(**Y**), i.e., the output of the feature extraction operation. After $n$ times of recurring nonlinearity mapping, the output is defined as $F_{n+1}$(**Y**${}_{n+1}$).

In nonlinear mapping, the size of $F_{i}$(**Y**${}_{i}$) must be identical with that of $F_{i+1}$(**Y**${}_{i+1}$) because $F_{i}$(**Y**${}_{i}$) is the output of the $i$th iteration and the input of the ($i+1$)th iteration. The output size of the convolution operation is calculated as ‘*output size* = (*input size* − $w_{2} + 2\cdot $ *zero-padding size*)/*stride* + 1*.*’ Here, $w_{2}$ is the size of $W_{i+1}$, and we set ‘*stride*’ to ‘1’ to fully utilize the extracted features. For this, the nonlinear mapping must have $3\times 3$ pixels $W_{i+1}$ with 1 pixel zero-padding. Obviously, more iterations lead to higher computational complexity. Accordingly, the time to process the resolution-enhanced MRI slices would be longer. Thus, selecting the appropriate $n$ is important to compromise between image quality and processing time.

After the nonlinear mapping, we obtain HR features using the convolution operation as Eq. (5) where $i$ equals $n+1$. The number of $W_{n+2}$ is set to $N_{feature}$. Then, we use a deconvolution operation to upscale and aggregate the HR features depending on $f$ as follows:

\begin{equation*}F_{n+3} \left ({{{\mathbf { Y}}_{n+3}} }\right)=Deconv\;\left ({{{\mathbf { Y}}_{n+3},W_{n+3},B_{n+3},f,p} }\right),\tag{6}\end{equation*}

where *Deconv* ($\cdot $) denotes the deconvolution function, $W_{n+3}$ indicates filters, $B_{n+3}$ represents biases of the reconstruction operation, and $p$ denotes zero-padding size. Here, the size of $W_{n+3}$, is $w_{n+3}$. In Eq. (6), **Y**${}_{n+3}$ equals $F_{n+2}$(**Y**${}_{n+2}$), i.e., the obtained HR features after the nonlinearity mapping operation. We summarize the proposed ACNS as the following pseudocode.

**Autoencoder-Inspired Convolutional Network-Based Super-Resolution (ACNS)**
Input: **Y**: Low-Resolution (LR) MRI
$f$: Scaling factor
$n$: Calculation iteration number of nonlinear mapping $\Theta $ = {$W_{1}$, $W_{2}$, $W_{3}$, $B_{1}$, $B_{2}$, $B_{3}$}: Convolutional network parameters
Output: $\hat { \boldsymbol {X}}$: estimated High-Resolution (HR) MRI
1) Extract features from Y with $\Theta $ by Eq. (4)
**for** $i=1$ to $n$ **do**
2) Map the extracted features of $F_{i +1}$($\textbf {Y}_{i +1}$) nonlinearly with $\Theta $ by Eq. (5)
**end for**
3) Acquire HR feature with $\Theta $ by Eq. (5)
4) Compute $\hat { \boldsymbol {X}}$ by up-scaling and aggregating HR features of $F_{n +3}$($\textbf {Y}_{n +3})$ with $\Theta $ by Eq. (6)

Training of ACNS is designed to find the optimal $\Theta $ that minimizes the loss between the estimated HR MRI slice $\hat {X}$, i.e., $F_{n+3}$(**Y**${}_{n+3}$), and the HR MRI slice **X**. We use Mean Squared Error (MSE) as the loss function

\begin{equation*}L\left ({\Theta }\right)=\frac {1}{2N}\sum \limits _{i=1}^{N} {\left \|{ {F\left ({{{\mathbf { Y}}^{i};f;\Theta } }\right)-{\mathbf { X}}^{i}} }\right \|^{2}},\tag{7}\end{equation*}

where $N$ indicates the number of the training samples. The loss function in Eq. (7) is minimized by the stochastic gradient descent algorithm based on backpropagation [71]. The weights are updated as follows:

\begin{equation*}\Delta _{i+1} =0.9\Delta _{i} -\eta \frac {\partial L(\Theta)}{\partial W_{i}^{l}},\quad W_{i+1}^{l} =W_{i}^{l} +\Delta _{i+1},\tag{8}\end{equation*}

where $l$ denotes a layer number, $\Delta _{i+1}$ is a current update value, $\Delta _{i}$ is a previous update value, $W^{l}_{i+1}$ is a current weight, $W^{l}_{i}$ is a previous weight, $\eta $ indicates a learning rate, and $\partial L(\Theta)/\partial W^{l}_{i}$ is a derivative of $L(\Theta)$. ACNS uses a Xavier algorithm for weight initialization to automatically determine initialization scale according to the network structure [71]. In [70], the authors showed that a combination of Xavier algorithm and PReLU function would either converge slowly or not converge when the network was very deep (i.e. 22 layers or deeper). However, the initialization method was empirically chosen considering the structure of ACNS that compromises image quality with computation time. In training, multiple local images were extracted as patches from both the HR and LR MRIs, and these patches were applied as the training input images.

The computation time of ACNS is determined by $N_{feature}$, $N_{map}$, $w_{1}$, $w_{n+3}$, and $n$. Obviously, the computational complexity increases with the number and size of the filters (i.e. $N_{feature}$, $N_{map}$, $w_{1}$ and $w_{n+3}$,) as well as the number of network layers (i.e. $n$). Furthermore, the selection of $N_{feature}$, $N_{map}$, $w_{1}$, $w_{n+3}$, $p$, and $n$ affects the image quality performance in addition to how well $\Theta $ (i.e. $W_{k}$ and $B_{k}$ where $k = 1, 2, \ldots, n+3$) is trained. Unlike computation time, it is impossible to grasp the explicit relationship between the network structure and its performance without validation. Therefore, the selection of $N_{feature}$, $N_{map}$, $w_{1}$, $w_{n+3}$, $p$, and $n$ is significant, and we define them as network parameters of ACNS. In designing ACNS, there is no restriction on choosing $N_{feature}$, $N_{map}$, and $n$. However, we selected $w_{1}$, $w_{n+3}$, and $p$ to satisfy the following four conditions:

1) $w_{1} < $ *LR patch size*,
2) $2p < f\cdot $ (*LR patch size* - $w_{1}$),
3) $p < w_{n+3}$, and
4) *LR patch size* $\le f~\cdot $ (*LR patch size* − $w_{1}) +\,\,\text{w}_{n+3} - 2 p \le f \cdot $ *LR patch size* + mod(*LR patch size*, 2),

where *Condition2* and *Condition4* are derived by constraints on the output size of the deconvolution operation: ‘*output size* = *stride* $\cdot $(*input size* − 1) $+ w_{n+3}$ − $2p$).’ Here, $f$ is assigned as ‘*stride*’, and ‘*input size*’ corresponds to ‘*output size*’ of the non-linear mapping layer, i.e., ‘*LR patch size* − $w_{1} +1$.’ Evaluation of image quality and processing time according to the network parameters is given in Section IV.

For the image quality evaluation, we used typical metrics in SR studies: Peak Signal-to-Noise Ratio (PSNR), Structure SIMilarity index (SSIM), and Information Fidelity Criterion (IFC) [9], [72]. PSNR was computed as

\begin{equation*}PSNR=20\log _{10} \left ({{MAX/MSE} }\right),\tag{9}\end{equation*}

where *MAX* is the maximum intensity value.

SSIM was calculated as

\begin{equation*}SSIM\left ({{x,y} }\right)=\frac {\left ({{2\mu _{x} \mu _{y} +C_{1}} }\right)\left ({{2\sigma _{xy} +C_{2}} }\right)}{\left ({{\mu _{x}^{2} +\mu _{y}^{2} +C_{1}} }\right)\left ({{\sigma _{x}^{2} +\sigma _{y}^{2} +C_{2}} }\right)},\tag{10}\end{equation*}

where $x$ corresponds to the MRIs enlarged by ACNS, $y$ indicates the ground truth MRIs, and $\mu _{x}$, $\mu _{y}$, $\sigma _{x}$, $\sigma _{y}$, and $\sigma _{xy}$ are means, variances, and covariance of $x$ and $y$, respectively. In *SSIM*, $C_{1}$ and $C_{2}$ denote stabilization constants calculated as $C_{1}=(K_{1}$ *MAX*)^2^ and $C_{2}=(K_{2}$ *MAX*)^2^, where $K_{1}\ll 1$ and $K_{2}\ll 1$. Here, we set $K_{1}$ as 0.1 and $K_{2}$ as 0.3 according to the Image Processing Toolbox of MATLAB.

IFC was calculated as

\begin{equation*}IFC=\sum \limits _{k\in subbands} {I\left ({{C^{N_{k},k};D^{N_{k},k}\left |{ {s^{N_{k},k}} }\right.} }\right)},\tag{11}\end{equation*}

where $C^{Nk,k}$, $D^{Nk,k}$, and $s^{Nk,k}$ denote $N_{k}$ coefficient from the reference image $C^{k}$, the test image $D^{k}$, and the random field of positive scalars $s^{k}$ of the $k$th sub-band, respectively.

## IV. Results

### A. Experimental Data

Virtual phantom and MRI data were used in the experiments. We obtained 200 images from a virtual model of the human torso with cardiac and respiratory motions, the 4D extended Cardiac-Torso (XCAT) Phantom [73]. The size of the virtual phantom model is $256\times 256\times201$ voxels, and the size of the acquired virtual phantom images were $200\times200$ pixels. Out of 713 slices, i.e. 256 coronal, 256 sagittal, and 201 transverse slices, we selected the slices containing a lung region only. Dataset1 contains 60 coronal slices. Dataset2 contains 60 sagittal slices. Dataset3 contains 60 transverse slices.

Dynamic multislice 2D True Fast Imaging with Steady state Precession (TrueFISP) and GRadient And Spin Echo (GRASE) images were collected from four volunteers using a ViewRay 0.35 T MRIgRT at the Washington University in St. Louis after volunteers provided informed consent. Table 3 describes the MRI data of the four volunteers used for performance verification of the proposed image SR method.

**TABLE 3:** MRI Data of Four Subjects for Performance Validation

Volunteer ID	Data #	View	Field of view (mm)	Slice thickness (mm)	# of images (slices $\times $ sets)	Slice size (pixel^2^)	Image protocol	Acquisition Type	Acquisition Time	BW
1	1	Sagittal	$300\times300$	5	$10\times10$	$128\times128$	TrueFISP	2D	289 ms/image	501 Hz/pixel
	2	Sagittal	$300\times300$	5	$10\times80$	$128\times128$	TrueFISP	2D	289 ms/image	501 Hz/pixel
2	3	Transverse	$300\times300$	5	$14\times20$	$128\times128$	TrueFISP	2D	438 ms/image	558 Hz/pixel
	4	Transverse	$281\times300$	5	$14\times20$	$128\times120$	TrueFISP	2D	173 ms/image	1002 Hz/pixel
3	5 to 8	Transverse	$340\times340$	6	$12\times6$	$64\times64$	GRASE	3D	600 ms/volume	2003 Hz/pixel
	9 & 10	Transverse	$340\times340$	6	$12\times60$	$64\times64$	GRASE	3D	600 ms/volume	2003 Hz/pixel
4	11 to 13	Coronal	$350\times350$	5	$10\times30$	$64\times64$	TrueFISP	3D	0.7 s/volume	898 Hz/pixel
	14 to 18	Sagittal	$350\times350$	5	$10\times30$	$64\times64$	TrueFISP	3D	0.7 s/volume	898 Hz/pixel
	19 & 20	Sagittal	$350\times350$	5	$20\times30$	$64\times64$	TrueFISP	3D	1.27 s/volume	898 Hz/pixel
	21 & 22	Coronal	$350\times350$	5	$20\times30$	$64\times64$	TrueFISP	3D	1.27 s/volume	898 Hz/pixel

Data1 and 2 are the sagittal MRIs from Volunteer1 and their image size is $128\times128$ pixels. Data3 and 4 are from the transverse MRIs from Volunteer2 and their image sizes are $128\times128$ pixels and $128\times120$ pixels, respectively. Data5 to 10 are the transverse MRIs from Volunteer3 and the image size is $64\times64$ pixels. Data11 to 22 are the coronal and sagittal MRIs from Volunteer4 with the size of $64\times64$ pixels. We additionally used 42 slices—14 coronal, 14 sagittal, and 14 transverse images—of $128\times128$ pixels from Volunteer3 and 5 slices—2 coronal, 2 sagittal, and 1 transverse images—of $128\times128$ pixels from Volunteer4 for the purpose of training the ACNS.

### B. Training

The original images were used as the ground truth in the training step. LR images were generated from the original images following the observation model of Eq. (1). The training datasets included 91 non-medical images [52], 50 XCAT images, and 42 MRIs.

There are two main reasons why we included non-medical images and XCAT images. First, ACNS estimates pixel loss from HR image to LR image, independent of MRI contrast properties. ACNS’s learning, i.e., mapping between LR and HR images in the image domain, depends on the observation model of (1). In this paper, we focus on the LR problem only. Other problems of MRI were not considered when defining our observation model. For example, ACNS does not address MRI distortion. Therefore, all the training datasets do not need to be MRI as long as the image pairs satisfy the relationship of (1). Second, the use of higher-resolution images in training leads to better image quality. The SR result would be able to reach the ground truth in an ideal case. Therefore, the resultant image quality is limited by the ground truth employed in training. The deep learning network trained by higher-resolution image sets can learn more details of the pixel information to be recovered. Accordingly, it is beneficial to include non-medical images, commonly used in computer vision studies, and pixelated XCAT images with highly clear boundaries in the training dataset because their resolution is higher than that of MRIs.

Since our maximum scaling factor $f$ is ‘4,’ the LR image size would be extremely small, only $16\times16$ pixels, if we selected the MRIs of $64\times64$ pixels for the training samples. Thus, we chose relatively larger matrix sizes among the TrueFISP images we collected: 42 MRIs of $128\times128$ pixels for training and 5 MRIs of $128\times128$ pixels for testing the training step. We produced the training images by splitting the training images into patches of $11\times11$ pixels with stride 4. The number of the patches was calculated as *floor* ((*LR image size* − *patch size* + 1)/ *stride*). For example, the LR MRIs of $64\times64$ pixels are generated by Eq. (1) when $f$ is 2 and 196 patches were created. Similarly, the ground truth images, i.e. the original MRIs, were also separated into local patches. Their patch size depends on the network parameters and the details are given in the following Section IV-*C*.

Our experiments were conducted on a PC with Intel®Xeon®CPU (ES-2637 3.50 GHz), NVIDIA Quadro M6000 24GB GPU, and 128 GB RAM. The ACNS model was trained using the Caffe package [74]. We set $\eta $ of feature extraction and nonlinear mapping layers to 0.001 and $\eta $ for the ACNS reconstruction layer to 0.0001. We denote the ACNS according to the network parameters, such as ACNS($N_{feature}$, $N_{map}$, $w_{1}$, $w_{n+3}$, $p$, $n$). For ACNS(16, 8, 5, 9, 5, 4), it took approximately 6 hours and 30 mins for training 183 images (91 non-medical images, 50 XCAT images, and 42 MRIs) with 1,000,000 training iterations.

We compared the performance of ACNS with existing CNN-based image SR methods—SRCNN, FSRCNN, and DRCN—their codes are available at [75], [76], and [77], respectively. Whereas SRCNN and FSRCNN codes are public, the authors of DRCN provided the trained network code only. In addition, we speculate that the authors of SRCNN and FSRCNN did not disclose all the details about how they trained and tuned the algorithm. Therefore, we used SRCNN and FSRCNN trained by its authors with superior performance than the ones trained by us.

The SRCNN model was trained by C. Dong *et al.* with 395,909 images from the ILSVRC 2013 ImageNet detection training partition, using cuda-convnet package [78]. According to [9], C. Dong *et al.* required three days on a machine with GTX 770 GPU, Intel CPU 3.10 GHz, and 16 GB memory to train the SRCNN model with 91 images. For tests, they took 0.14 s to process an image of $288\times288$ pixels. The FSRCNN model was also trained by C. Dong *et al.* with the Caffe package. They trained the FSRCNN model based on 91 images first, then fine-tuned it with 100 images. The training image sizes were from $131\times112$ pixels to $710\times704$ pixels. The DRCN model was trained by J. Kim *et al.* using the MathConvNet package [79]. The maximum number of recursions of the inference network was 16. Their training time was six days on a machine with a Titan X GPU, and it took 1 s to process an image of $288\times288$ pixels.

### C. Network Parameter Selection

The network parameters—$N_{feature}$, $N_{map}$, $w_{1}$, $w_{n+3}$, $p$, and $n$—decide not only the network architecture, but also the image quality performance and computation speed of ACNS. To find optimal network parameters, we created various ACNS models for $f = 2$ with different network parameter values and trained them on the training settings described in Section IV-*B*.

**FIGURE 2.**
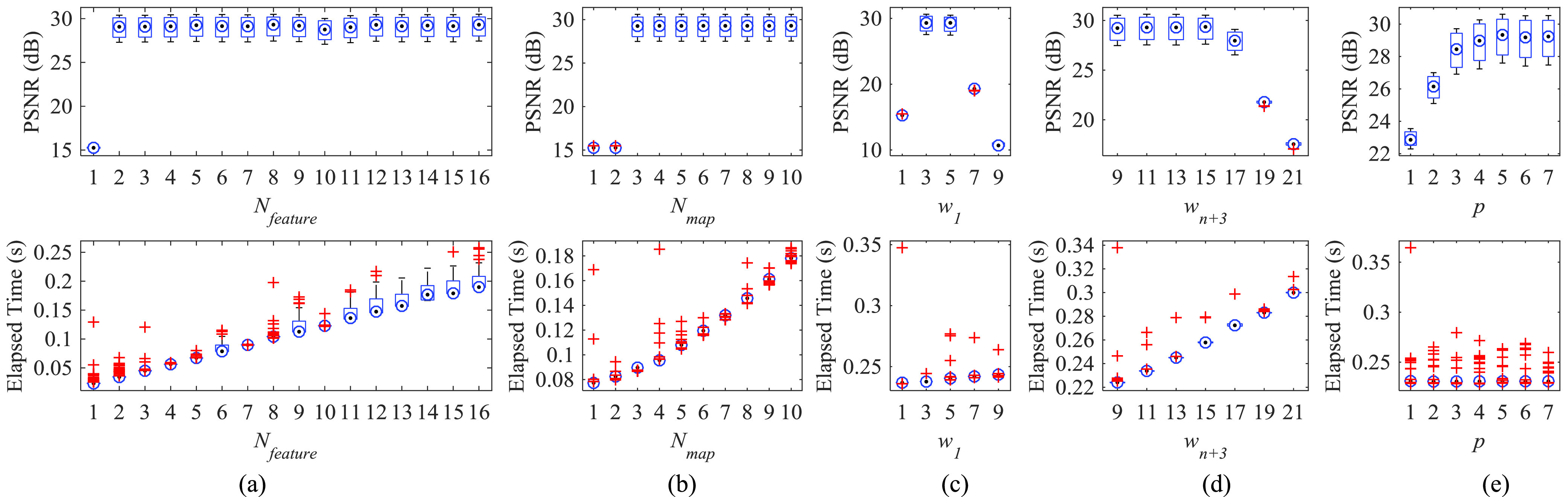
PSNR and elapsed time according to: (a) the number of filters in feature extraction $N_{feature}$, (b) the number of filters in the nonlinear mapping $N_{map}$, (c) the filter size in the feature extraction $w_{1}$, (d) the filter size in reconstruction $w_{n +3}$, and (e) the zero-padding size $p$, when $f=2$.

Figs. 2 (a)-(e) show the comparison of the box plot results from tests by ACNS($N_{feature}$, 4, 5, 11, 5, 4), ACNS(7, $N_{map}$, 3, 11, 7, 4), ACNS(20, 4, $w_{1}$, 11, 10-$w_{1}$, 4), ACNS(20, 4, 3, $w_{n+3}$, 7, 4), and RDLS(20, 4, 3, 9, $p$, 4) for 100 MRIs from Dataset1, respectively. The red ‘+’ symbols in Fig. 2 represent outliers. The first row is PSNR and the second row is elapsed time results in Fig. 2. Whereas the ranges of $w_{1}$, $w_{n+3}$, and $p$ are dependent on the LR and HR patch sizes, $N_{feature}$, $N_{map}$, and $n$ can be any natural number. We first compared the ACNS models with different $N_{feature}$ and $N_{map}$ values. As shown in Figs. 2 (a) and (b), PSNR results were relatively high and stable when $N_{feature}$ was ≥2 and $N_{map}$ was ≥3. In addition, the larger $N_{feature}$ and $N_{map}$ took longer to process the SR image.

Candidate values of $w_{1}$, $w_{n+3}$, and $p$ must satisfy the ACNS design conditions explained in Section III. With the LR patch size of 11, the conditions are simplified: *Condition*1: $w_{1} < 11$; *Condition*2: $p < 11$ - $w_{1}$; *Condition*3: $p < w_{n +3}$; and *Condition*4: $11\le22$ - $2w_{1} +\,\,\text{w}_{\mathrm {n+3}}$ - $2p \le23$. Specifically, the candidate set for $w_{1}$ is {1, 3, 5, 7, 9} by *Condition*1, $p$ is {0, 1, $2\ldots 9$} by *Condition*2, and $w_{n +3}$ is {1, 3, $5\ldots 21$} by *Condition4*. Any candidate for $w_{1}$ can be selected regardless of any given $w_{n +3}$ and $p$ since $w_{1}$ is an independent variable. However, selection of $p$ and $w_{n +3}$ relies on $w_{1}$. A total of 167 networks are satisfied with the ACNS design conditions. As shown in Figs. 2 (c)-(e), larger $w_{n +3}$, and $w_{1}$ had a subtle increment in the elapsed time, but there was no elapsed time change according to $p$ on average. On the other hand, PSNR was above 29 dB when $w_{1}$ was 3 and 5, and $w_{n +3}$ was less than 17. Besides, PSNR was over 29 dB at $p$ closer to its maximum value, i.e. 10 - $w_{1}$, as shown in Figs. 2 (c)-(e). As a result, we chose a $w_{1}$ of 5, $w_{n +3}$ of 9, and $p$ of 10 - $w_{1}$ (i.e. 5) based on the results in Fig. 2.

**FIGURE 3.**
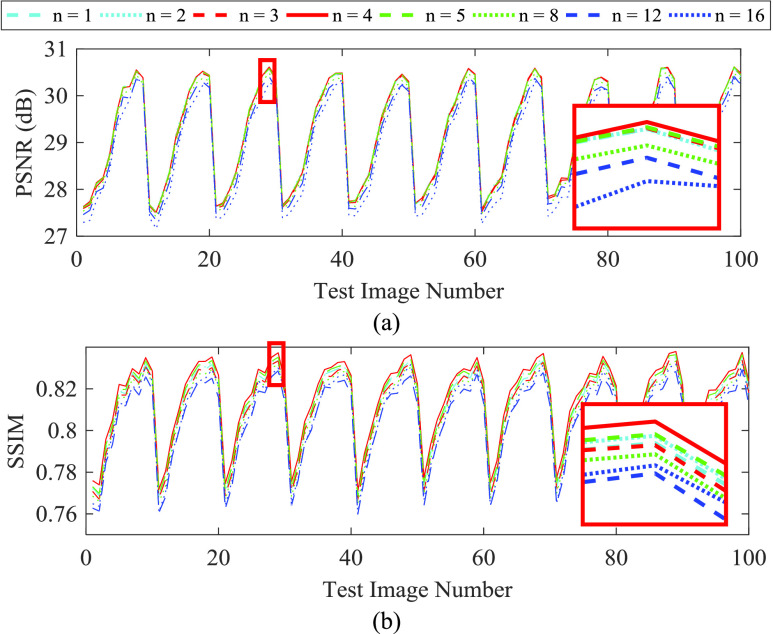
Image quality performance of ACNS according to the number of nonlinear mapping layers $n$: (a) PSNR and (b) SSIM results. A cyan dashed, cyan dotted, red dashed, red solid, green dashed, green dotted, blue dashed, and blue dotted lines correspond to $n$ of 1, 2, 3, 4, 5, 8, 12, and 16, respectively. For visibility, we magnified a region with a red rectangle.

Fig. 3 shows the image quality performance of ACNS according to the number of nonlinear mapping recursion $n$. The PSNR and SSIM results are shown in Fig. 3. ACNS did not produce a distinct difference according to $n$. Interestingly, both PSNR and SSIM were not improved with more recursion of the nonlinear mapping, whereas elapsed time increased from 0.22s to 0.45s. From the results in Fig. 3, we determined $n$ as 4. The cyclic shape of the results in Fig. 3 represent physiologic motion sampled during the test dataset acquisition. The dataset consisted of multislice single-shot TrueFISP 2D acquisitions with 10 slices/volume. Each slice was acquired in 0.3 s and sampled at 0.33 Hz (i.e. 3 s/volume).

**FIGURE 4.**
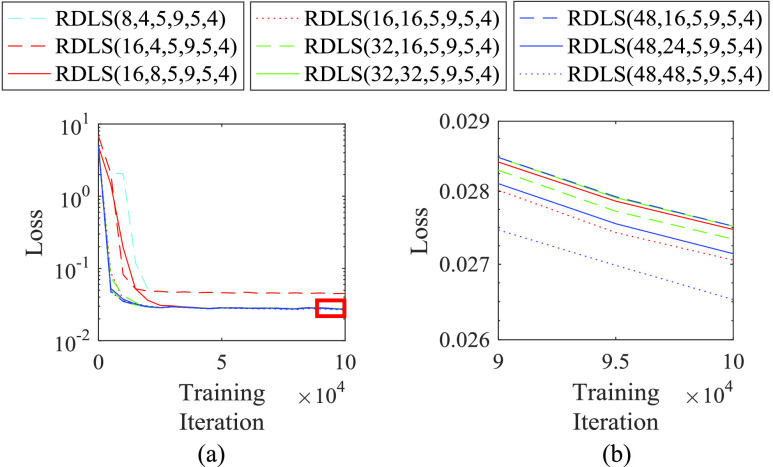
Training loss of ACNS with various combinations of $N_{feature}$ and $N_{map}$; the designated $w_{1}$, $w_{n +3}$, and $p$; and $n$ of 4. The area marked with a red rectangle in (a) is magnified as (b). A y-axis, i.e. loss, is in a log scale. A cyan dashed line is the result of ACNS(8, 4, 5, 9, 5, 4), a red dashed line is the result of ACNS(16, 4, 5, 9, 5, 4), a red solid line is the result of ACNS(16, 8, 5, 9, 5, 4), a red dotted line is the result of ACNS(16, 16, 5, 9, 5, 4), a green dashed line is the result of ACNS(32, 16, 5, 9, 5, 4), a green solid line is the result of ACNS(32, 32, 5, 9, 5, 4), a blue dashed line is the result of ACNS(48, 16, 5, 9, 5, 4), a blue solid line is the result of ACNS(48, 24, 5, 9, 5, 4), and a blue dotted line is the result of ACNS(48, 48, 5, 9, 5, 4).

Although we found appropriate ranges of $N_{feature}$ and $N_{map}$ from Figs. 2 (a) and (b), we conducted further experiments to investigate a relationship between a combination of $N_{feature}$ and $N_{map}$ and the image quality performance. Because the training in ACNS strains to minimize the loss of the ACNS, i.e. Eq. (7), training loss can be translated as image quality performance. Fig. 4 illustrates the training loss of ACNS with various combinations of $N_{feature}$ and $N_{map}$; the designated $w_{1}$, $w_{n+3}$, and $p$; and $n$ of 4. We presented Fig. 4 with a log scale for loss on the y-axis, to show delicate loss differences between the ACNS networks according to the combination of $N_{feature}$ and $N_{map}$.

As shown in Fig. 4 (a), ACNS(8, 4, 5, 9, 5, 4) and ACNS(16, 4, 5, 9, 5, 4) had relatively large training losses compared to the other ACNS networks. In the results of Fig. 4 (b), ACNS(48, 48, 5, 9, 5, 4) showed the lowest training loss. However, the training loss of ACNS(16, 16, 5, 9, 4) was less than that of ACNS(16, 16, 5, 9, 5, 4), ACNS(32, 16, 5, 9, 5, 4), ACNS(32, 32, 5, 9, 5, 4), ACNS(48, 16, 5, 9, 5, 4), and ACNS(48, 24, 5, 9, 5, 4). This means that there is no significant difference of the image quality performance between the ACNS networks with $N_{feature}$ and $N_{map}$, above the values 8 and 4, respectively, according to the result of Fig. 4. Therefore, we selected $N_{feature}$ as 16 and $N_{map}$ as 8 considering the computation speed of ACNS. In the remaining Section IV, we used ACNS(16, 8, 5, 9, 5, 4) for all $f\text{s}$ according to the results in this subsection. The HR patch size was determined as 11 for $f=2$, 17 for $f=3$, and 23 for $f=4$.

### D. Image Quality Evaluation

We verified the image quality performance of ACNS by comparing it with SRCNN, FSRCNN, and DRCN. Unlike ACNS and FSRCNN, SRCNN and DRCN improve image resolution while maintaining the original image size, without image size expansion depending on $f$. For comparisons with the identical input images, we first enlarged the LR image using bicubic upsampling according to $f$, then applied the upsampled LR image to SRCNN and DRCN, whereas the LR image was directly used as the input of ACNS and FSRCNN. We used the original images as the ground truth and the LR images produced by Eq. (1) to measure PSNR, SSIM, and IFC. The ground truth images need to be large enough for the maximum $f$, as in training. Therefore, three XCAT datasets, and Volunteer1 and Volunteer2 datasets were used for PSNR, SSIM, and FIC comparison.

**FIGURE 5.**
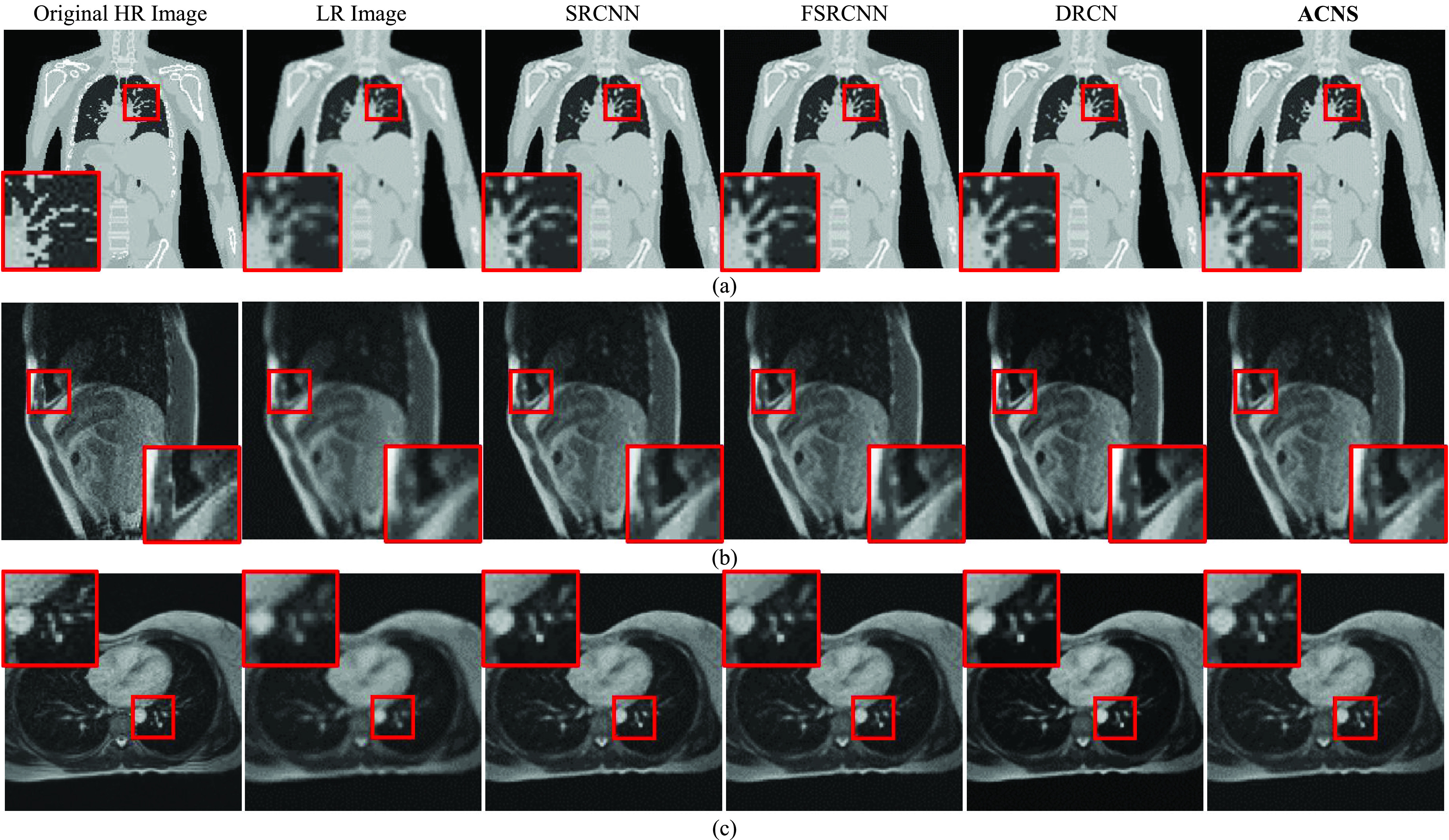
Comparison of resolution-enhanced XCAT image and MRIs using SRCNN, FSRCNN, DRCN, and ACNS: (a) XCAT dataset1, (b) Volunteer1, and (c) Volunteer2 datasets. The first and second columns present the original HR and LR images; and the third, fourth, and fifth columns indicate SR results from SRCNN, FSRCNN, and DRCN, respectively. The sixth column is the resolution-enhanced XCAT image and MRIs by ACNS. All results were at a $f$ of 2. To provide visible comparison, the images were partially enlarged from the regions marked as red squares.

Fig. 5 shows a comparison of the resolution-enhanced XCAT image and MRIs. We randomly selected three resultant images from the XCAT dataset1, Volunteer1 and Volunteer2 datasets, shown in Figs. 5 (a), (b), and (c), respectively. As shown in Fig. 5, all resultant images were more detailed than the LR XCAT image and LR MRIs. SRCNN, FSRCNN, and ACNS maintained the image intensity values regardless of resolution improvement. However, DRCN increased the intensity contrast of the MRIs.

In Table 4, we provided the quantitative results, i.e., average PSNR, SSIM, and IFC of the resolution-enhanced virtual phantom images. The best results are highlighted in bold font. The average PSNRs of three XCAT datasets for three $f\text{s}$ using SRCNN, FSRCNN, DRCN, and ACNS were 26.36 dB, 25.96 dB, 26.73 dB, and 26.53 dB, the average SSIMs were 0.86, 0.48, 0.93, and 0.84, and the average IFCs were 2.32, 2.38, 2.45, and 2.50, respectively. As shown in Table 4, ACNS showed the best IFC performance and the second best PSNR performance next to DRCN. In addition, ACNS had better performance than FSRCNN in terms of SSIM.

**TABLE 4:** Average PSNR, SSIM, and IFC of Resolution-enhanced Virtual Phantom Images

Metric	Data-set	Scale	SRCNN [10]	FSRCNN [11]	DRCN [12]	ACNS
PSNR (dB)	1	$\times 2$	26.70 ± 1.55	26.54 ± 1.27	24.40 ± 1.38	27.01 ± 1.50
		$\times 3$	25.68 ± 2.25	24.56 ± 1.33	25.21 ± 1.34	24.80 ± 1.38
		$\times 4$	24.10 ± 1.97	23.29 ± 1.28	27.49 ± 1.23	23.86 ± 1.35
	2	$\times 2$	28.52 ± 0.82	28.03 ± 0.71	25.67 ± 1.07	28.84 ± 0.96
		$\times 3$	26.41 ± 0.88	26.17 ± 0.78	26.31 ± 1.10	26.73 ± 0.98
		$\times 4$	25.01 ± 0.92	24.97 ± 1.28	29.19 ± 0.93	25.85 ± 1.04
	3	$\times 2$	28.87 ± 1.55	28.36 ± 0.57	25.58 ± 0.88	29.10 ± 0.70
		$\times 3$	26.66 ± 2.25	26.53 ± 0.64	26.69 ± 0.66	26.74 ± 0.71
		$\times 4$	25.28 ± 1.97	25.15 ± 1.28	30.05 ± 0.84	25.88 ± 0.73
SSIM	1	$\times 2$	0.88 ± 0.04	0.63 ± 0.10	0.88 ± 0.03	0.83 ± 0.01
		$\times 3$	0.84 ± 0.07	0.59 ± 0.07	0.91 ± 0.03	0.82 ± 0.05
		$\times 4$	0.77 ± 0.09	0.54 ± 0.06	0.93 ± 0.01	0.76 ± 0.06
	2	$\times 2$	0.92 ± 0.01	0.47 ± 0.02	0.92 ± 0.01	0.84 ± 0.01
		$\times 3$	0.87 ± 0.02	0.46 ± 0.01	0.93 ± 0.01	0.88 ± 0.02
		$\times 4$	0.82 ± 0.02	0.43 ± 0.06	0.95 ± 0.01	0.85 ± 0.02
	3	$\times 2$	0.93 ± 0.04	0.42 ± 0.02	0.93 ± 0.01	0.84 ± 0.00
		$\times 3$	0.89 ± 0.07	0.42 ± 0.02	0.94 ± 0.01	0.89 ± 0.01
		$\times 4$	0.85 ± 0.09	0.39 ± 0.06	0.96 ± 0.01	0.86 ± 0.01
IFC	1	$\times 2$	4.34 ± 0.78	4.44 ± 0.81	3.37 ± 0.67	4.77 ± 0.89
		$\times 3$	2.80 ± 0.40	2.92 ± 0.48	3.07 ± 0.75	3.01 ± 0.53
		$\times 4$	1.89 ± 0.26	1.93 ± 0.30	3.00 ± 0.72	2.08 ± 0.34
	2	$\times 2$	2.92 ± 0.23	2.93 ± 0.24	2.22 ± 0.18	3.19 ± 0.23
		$\times 3$	1.88 ± 0.13	1.98 ± 0.15	2.24 ± 0.19	2.07 ± 0.13
		$\times 4$	1.32 ± 0.09	1.33 ± 0.30	2.16 ± 0.15	1.46 ± 0.09
	3	$\times 2$	2.66 ± 0.78	2.67 ± 0.23	1.93 ± 0.20	2.76 ± 0.25
		$\times 3$	1.80 ± 0.40	1.89 ± 0.17	2.04 ± 0.21	1.81 ± 0.16
		$\times 4$	1.29 ± 0.26	1.30 ± 0.30	1.99 ± 0.19	1.32 ± 0.11

In the comparison of Table 4, there was an interesting finding that the average SSIM results by FSRCNN were exceedingly inferior to ACNS, DRCN, and even SRCNN with the simplest layer structure. The virtual phantom images contain the background along with the human torso. The background area is large relative to the virtual phantom image, and the background’s intensity value is ‘0.’ During the resolution improvement, FSRCNN failed to preserve the intensity values in the background while SRCNN, DRCN, and ACNS did. The intensity values of the background in the resolution-enhanced images by FSRCNN varied between ‘2’ and ‘7.’ The virtual phantom image was stored with 8 bits per pixel i.e., intensity ranges between ‘0’ and ‘255.’ The failure of FSRCNN in conserving the background intensity values degraded its SSIM results. The inferior SSIM results of FSRCNN were not observed in other studies using the natural images because the majority of natural images commonly used in computer vision do not include large background areas.

Table 5 compares average PSNR, SSIM, and IFC of the resolution-enhanced MRIs. The best results are highlighted using bold font. The average PSNR, SSIM, and IFC of the MRIs were greater at the smaller $f$. The average PSNRs of two volunteer datasets for three $f\text{s}$ using SRCNN, FSRCNN, DRCN, and ACNS were 28.72 dB, 28.96 dB, 29.26 dB, and 29.25 dB, the average SSIMs were 0.76, 0.77, 0.77, and 0.80, and the average IFCs were 3.81, 4.09, 4.35, and 3.85, respectively.

**TABLE 5:** Average PSNR, SSIM, and IFC of Resolution-Enhanced MRIs

Metric	Volunteer	Scale	SRCNN [10]	FSRCNN [11]	DRCN [12]	ACNS
PSNR (dB)	1	$\times 2$	29.62 ± 0.76	29.79 ± 0.78	29.84 ± 0.75	29.58 ± 0.88
		$\times 3$	29.21 ± 2.50	29.40 ± 2.48	29.67 ± 2.59	27.88 ± 0.98
		$\times 4$	27.22 ± 2.10	27.54 ± 2.12	27.98 ± 1.95	26.38 ± 1.09
	2	$\times 2$	30.91 ± 2.27	31.10 ± 2.26	31.31 ± 2.54	33.12 ± 2.11
		$\times 3$	28.62 ± 1.78	28.86 ± 1.87	29.18 ± 1.78	30.37 ± 2.07
		$\times 4$	26.71 ± 1.58	27.05 ± 1.62	27.59 ± 1.49	28.15 ± 1.48
SSIM	1	$\times 2$	0.80 ± 0.02	0.81 ± 0.02	0.81 ± 0.02	0.82 ± 0.02
		$\times 3$	0.77 ± 0.07	0.78 ± 0.07	0.78 ± 0.07	0.75 ± 0.02
		$\times 4$	0.70 ± 0.07	0.71 ± 0.07	0.72 ± 0.07	0.71 ± 0.03
	2	$\times 2$	0.84 ± 0.05	0.84 ± 0.05	0.84 ± 0.05	0.91 ± 0.03
		$\times 3$	0.76 ± 0.06	0.76 ± 0.06	0.77 ± 0.06	0.84 ± 0.04
		$\times 4$	0.69 ± 0.06	0.70 ± 0.06	0.72 ± 0.06	0.79 ± 0.04
IFC	1	$\times 2$	5.19 ± 0.15	5.56 ± 0.15	5.79 ± 0.17	5.58 ± 0.12
		$\times 3$	4.21 ± 1.05	4.53 ± 1.09	4.78 ± 1.14	3.48 ± 0.09
		$\times 4$	2.67 ± 0.62	2.86 ± 0.70	3.14 ± 0.64	2.32 ± 0.11
	2	$\times 2$	5.33 ± 0.30	5.70 ± 0.30	5.99 ± 0.40	5.94 ± 0.35
		$\times 3$	3.33 ± 0.13	3.62 ± 0.16	3.84 ± 0.14	3.52 ± 0.22
		$\times 4$	2.15 ± 0.11	2.24 ± 0.11	2.58 ± 0.12	2.27 ± 0.11

ACNS achieved the highest average PSNR of 33.12 dB and SSIM of 0.91 for the Volunteer2 dataset and $f$ of 2 as shown in Table 5. ACNS mostly outperformed SRCNN, FSRCNN, and DRCN regarding PSNR and SSIM in the experiment using the Voluneer2 dataset. IFC results of ACNS was relatively low compared with FSRCNN and DRCN. For the Volunteer1 dataset, the performance of DRCN was slightly better than the other methods.

**FIGURE 6.**
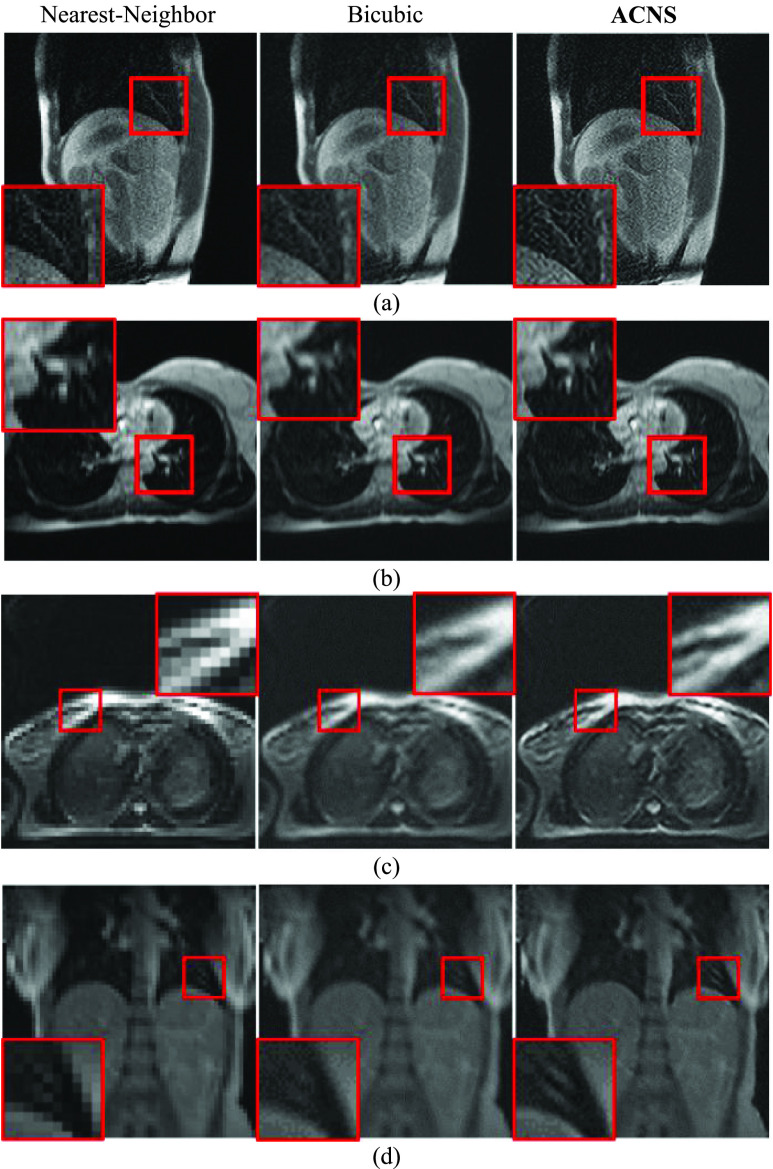
MRIs enlarged by ACNS: (a) Volunteer1, (b) Volunteer2, (c) Volunteer3, and (d) Volunteer4 datasets. Each MRI was randomly selected. The first and second columns are the original MRIs magnified by nearest-neighbor interpolation and bicubic upsampling, and the third column is the resulting MRIs from ACNS, at a $f$ of 3. We magnified the area in the red square to show more details.

Fig. 6 shows MRIs of Volunteer1, Volunteer2, Volunteer3, and Volunteer4 datasets, enlarged by nearest-neighbor interpolation, bicubic interpolation, and ACNS. To obtain the enlarged MRIs as our ultimate goal, we used the original MRIs as the inputs of ACNS. As shown in Fig. 6, MRIs acquired by ACNS were less blurry than MRIs by bicubic upsampling and less pixelated than nearest-neighbor interpolation. In Fig. 6 (d), the MRIs enlarged by nearest-neighbor interpolation and bicubic upsampling lost pixel information of two bright lines at the center of the red square after its size changed while the ACNS images maintained it. However, ACNS accentuated artifacts as illustrated in Fig. 6 (a) and (c). This is because ACNS does not selectively remedy missing pixel information.

### E. Computational Time Comparison

We compare the running times to produce resolution-enhanced images using SRCNN, FSRCNN, DRCN, and ACNS in Table 6. The best results are presented with bold font. For XCAT datasets, the input images of ACNS and FSRCNN were generated from the original images by Eq. (1) to obtain the resultant images of the identical size to the original images. For MRI datasets, we used the original images as the inputs of ACNS and FSRCNN, and upsampled the images with $f$ as the inputs of SRCNN and DRCN. The image size fed to the network varied and this caused a different level of computation for each method. ACNS and FSRCNN had $f^{2}$ times smaller input images than SRCNN and DRCN. Thus, we can observe diminishing patterns with $f$ in ACNS and FSRCNN results with $f$ for XCAT datasets and increasing patterns with $f$ in SRCNN and DRCN for MRI datasets in Table 6.

**TABLE 6:** Running Time

Dataset	Scale	SRCNN [10] (s)	FSRCNN [11] (s)	DRCN [12] (s)	ACNS(s)
XCAT 1	$\times 2$	1.59	1.96	9.06	0.47
	$\times 3$	1.55	1.16	9.06	0.26
	$\times 4$	1.58	0.89	9.30	0.19
XCAT 2	$\times 2$	1.53	1.97	9.16	0.47
	$\times 3$	1.54	1.14	9.13	0.26
	$\times 4$	1.56	0.87	9.11	0.19
XCAT 3	$\times 2$	1.55	1.97	9.22	0.47
	$\times 3$	1.53	1.14	9.14	0.26
	$\times 4$	1.54	0.88	9.29	0.19
Volunteer1	$\times 2$	2.20	2.51	14.10	0.71
	$\times 3$	6.92	2.49	29.68	0.71
	$\times 4$	11.45	2.62	56.79	0.75
Volunteer2	$\times 2$	2.20	2.41	14.95	0.69
	$\times 3$	6.43	2.43	30.72	0.70
	$\times 4$	11.55	2.57	56.95	0.74
Volunteer3	$\times 2$	0.99	0.92	3.85	0.25
	$\times 3$	1.51	0.93	9.44	0.25
	$\times 4$	2.18	0.94	15.63	0.25
Volunteer4	$\times 2$	0.97	0.93	3.78	0.25
	$\times 3$	1.48	0.92	8.98	0.25
	$\times 4$	2.18	0.94	15.50	0.25

As shown in Table 6, the average running time of ACNS was 0.71 s for the Volunteer1 and Volunteer2 datasets and 0.25 s for the Volunteer3 and Volunteer4 datasets. The test time measured in each method varied depending on the image size. The running time per $100\times100$ pixels of the resultant image was 0.89 s in SRCNN, 0.54 s in FSRCNN, 4.91 s in DRCN, and 0.14 s in ACNS. Accordingly, ACNS was 6, 4, and 35 times faster than SRCNN, FSRCNN, and DRCN, respectively. Although DRCN produced outstanding SR images, it required the longest computational time.

## V. Discussion

In the experiments, ACNS not only achieved comparable PSNR, SSIM, and IFC results to other CNN-based SR methods, but also substantially reduced the computational time compared to the methods currently considered state-of-the-art. The performance of ACNS results from its network structure. Given an identical training environment, the larger number of parameters, including the filters and layers, and bigger filter size caused longer computational time and did not lead to better image quality. This demonstrated that the common belief, “the deeper the better,” [22] is not always true. We determined the network structure of ACNS based on the experimental results in Section III-C. ACNS’s network structure is a compromise between image quality and computational time. ACNS consists of 6 layers and 5,728 parameters, whereas DRCN has 18 layers—when its recursive layers are unfolded—and 1,774,080 parameters. Thus, DRCN inevitably takes longer to calculate its outcome.

More complex and deeper networks require more painstaking and cumbersome training. Given two well-trained networks with different network depths, the deeper network (with higher nonlinearity) would be expected to achieve a greater improvement in image quality than the shallower network. Therefore, the well-trained DRCN yielded qualitatively better results than the other methods: SRCNN, FSRCNN, and ACNS.

SRCNN has only 3 layers, which is the shallowest layer structure. Therefore, SRCNN has less nonlinearity than FSRCNN, DRCN and ACNS, resulting in its inferior performance in image quality. Despite its simple layer structure, SRCNN generally demanded longer computational time than FSRCNN and ACNS due to its intra-layer design, i.e., the number and size of filters at each layer. Specifically, the number of the parameters was 8,032 for SRCNN and 12,464 for FSRCNN, i.e., 1.4 and 2.2 times greater than the number of the parameters in ACNS, respectively. We used the smallest practical number of the parameters in ACNS. We assume that the proposed framework can reduce the computational complexity and inference time due to fewer parameters and appropriate network structure than other methods.

The deconvolution operation also affected the computational time. Both ACNS and FSRCNN can produce an image of identical size with $f^{2}$ times less inputs than SRCNN and DRCN by using deconvolution for upsampling. Hence, the computation workload in ACNS and FSRCNN are $f^{2}$ times less than SRCNN and DRCN. We can enhance the MRI resolution using SRCNN and DRCN before upsampling to preserve the identical size of the inputs and resultant images. This would prevent a computational time increase proportional to $f^{2}$. However, the image quality would be appreciably deteriorated. The nearest-neighbor interpolation would unnaturally pixelate and the bicubic interpolation would blur the estimated HR MRI through upsampling. It is not practical to reduce computational time without a sacrifice in image quality.

The biggest problem in applying the current CNN-based SR methods to 4D MRI for MRIgRT is their long computational time. Since 4D MRI needs to be promptly obtained and used during the treatment, it is necessary to accomplish both high image quality and fast processing. Ideally, reconstruction and resolution improvement of 4D MRI should be performed in real-time. According to [11], when FSRCNN was implemented in C++, the average computational time of FSRCNN was 0.061s at $f$ of 3 for dataset *Set14*, which includes 14 images in a range of $276\times276$ pixels to $720\times576$ pixels. However, we implemented our method and FSRCNN in MATLAB, and the average test time of FSRCNN was 2.49 s at $f$ of 3, for Volunteer1 dataset with 900 MRIs of $128\times128$ pixels. For the same dataset and $f$, ACNS was 3 times faster than FSRCNN. Accordingly, ACNS is expected to achieve real-time processing in C++. Moreover, GPU performance has become very fast. The GPU used in the experiment can process 7 trillion floating point operations per second (TFLOP) and the performance of the NVIDIA Quadro RTX 8000, one of the state-of-the-art GPUs, is 16.3 TFLOP. Thus, the real-time processing is feasible with high-performance GPUs in combination with implementation using C++.

Additionally, our experiments showed the importance of network parameter selection in CNN-based SR. Based on the experimental results, the performance of ACNS did not rely on each network parameter, i.e., number and size of filters, zero-padding, and the number of layers, but their combination. Although more layers and filters led to longer computation time, they did not result in better image quality. We also observed that the training datasets affect the performance of ACNS. Therefore, there are no universal CNN-based SR methods. Instead, we need to customize the method for its purpose by empirically selecting optimal parameters and using appropriate training datasets. This evokes caution that when using deep learning methods for medical images, the performance with ensembles of the experimental data sets need to be continuously evaluated for their reliability because the deep learning methods are not transparent [20].

## VI. Conclusion

In this study we demonstrated ACNS, an in-plane SR method for MRI that recovers missing image information of LR MRIs by CNN-based nonlinear mapping between LR and HR features. Our experiments showed that ACNS achieved comparable image quality improvement as well as outstanding processing speed, which was approximately 6, 4, and 35 times faster than SRCNN, FSRCNN, and DRCN, respectively. The result implies the potential application of ACNS to real-time resolution enhancement of 4D MRI in MRIgRT. Additionally, we presented experimental analysis regarding the relationship between deep learning network parameters and the network’s performance. According to the experimental results, the deep learning-based SR method needs to be customized for its purpose through empirical selection of the optimal parameters and the use of appropriate training datasets.

In this study, we focused on only the in-plane resolution enhancement for MRI. However, there is a clinical demand for the through-plane resolution enhancement in gating based on 3D or 4D MRI. Therefore, our future work aims to enhance the through-plane resolution of 3D and 4D MRI.
